# Non-pharmacological interventions for behavioral and psychological symptoms of dementia: A systematic review and network meta-analysis protocol

**DOI:** 10.3389/fpsyt.2022.1039752

**Published:** 2022-11-29

**Authors:** Ya-Qin Li, Zi-Han Yin, Xin-Yue Zhang, Zheng-Hong Chen, Man-Ze Xia, Lai-Xi Ji, Fan-Rong Liang

**Affiliations:** ^1^School of Acu-Mox and Tuina, Chengdu University of Traditional Chinese Medicine, Chengdu, Sichuan, China; ^2^The 3rd Teaching Hospital, Shanxi University of Chinese Medicine, Jinzhong, China

**Keywords:** dementia, non-pharmacological treatment, behavioral and psychological symptoms of dementia (BPSD), randomized controlled trial, systematic review and meta-analysis

## Abstract

**Introduction:**

Dementia patients often experience behavioral and psychological symptoms (BPSD), which severely affect their quality of life and activities of daily living. Non-pharmacological interventions are effective in treating BPSD, according to multiple clinical trials and systematic reviews. However, the optimal non-pharmacological treatment remains controversial. Therefore, the study aims to evaluate and compare multiple non-pharmacological methods for treating BPSD in order to identify the optimal non-pharmacological intervention.

**Objective:**

This study aims to perform a systematic review and network meta-analysis of evidence on non-pharmacological interventions in the treatment of BPSD, which may potentially guide future research and clinical decisions.

**Methods:**

In order to select potentially relevant randomized controlled trials (RCTs), 10 academic databases and 3 clinical trial registries will be systematically searched from inception until the 1 October 2022. Two researchers will independently extract information from eligible articles. The primary outcome is the severity of BPSD. Herein, Pairwise and Bayesian network meta-analyses will be conducted utilizing STATA 15.0 and ADDIS 1.16.8. Evidence quality will be assessed using the Grading of Recommendations Assessment, Development, and Evaluation (GRADE).

**Results:**

Results from this study will be published in peer-reviewed journals or conference reports.

**Discussion:**

In this study, we aim to comparatively assess the efficacy of various non-pharmacological treatments for BPSD. Findings from this review will help clinicians to make evidence-based treatment decisions.

**Systematic review registration:**

[https://www.crd.york.ac.uk/prospero/], identifier [CRD42022352095].

## Introduction

### Rationale

Globally, approximately 50 million people are living with dementia, and the number is expected to reach 152.8 million by 2050 (1). Unfortunately, it has been found that a surprising number of individuals living with dementia do not receive any post-diagnosis support beyond the information they received at diagnosis (2). Indeed, in addition to the symptom of cognitive decline, individuals with dementia frequently manifest behavioral and psychological disturbances like agitation, depression, anxiety, apathy, and delusions, among others (3–5). It is estimated that 80–90% of patients with dementia develop behavioral and psychological symptoms of dementia (BPSD) during their illness (6). BPSD can have serious consequences, including prolonged hospitalization, increased health care costs and mortality (7–9), resulting in great suffering to patients as well as their families, putting a huge burden on society. The increased prevalence and incidence of BPSD underscores the need for effective treatment strategies. Therefore, it is imperative to explore effective pharmacological and non-pharmacological approaches to address BPSD.

Antipsychotics and anticonvulsants have demonstrated significant efficacy in the treatment of certain aspects of BPSD but are also associated with a higher rate of adverse events (10). In this context, pharmacological treatments are often used as a last resort in contrast to non-pharmacological therapies (11). Numerous non-pharmacological interventions have been used in managing BPSD, including physical exercise, cognitive training, music therapy, non-invasive brain stimulation, and acupuncture therapy, to name a few. A non-pharmacological approach could potentially reduce the severity of BPSD with comparable effectiveness to pharmacological treatment (12). Meanwhile, a recent study has shown that reminiscence, cognitive stimulation/rehabilitation, and other non-pharmacological approaches can reduce symptoms of depression in individuals with dementia (13). To date, most dementia management guidelines still recommend non-pharmacological intervention as the preferred first line of treatment. There is, however, a lack of consensus on the most effective non-pharmacological approach for BPSD, resulting in significant challenges in developing a definitive treatment protocol.

Network meta-analysis (NMA) is a statistical method that can synthesize direct and indirect evidence to rank different interventions from multiple trials in a network (14, 15). This type of analysis can provide more precise evidence for decision-makers to choose optimal treatment in clinical practice. To facilitate the selection of the most appropriate non-pharmacological treatment for BPSD patients, this study aims to perform an NMA that compares and ranks various non-pharmacological treatments for BPSD patients.

### Objectives

The purpose of this study is to evaluate the efficacy of non-pharmacological interventions in the treatment of BPSD and provide a clinical reference for health policy decision-makers.

## Methods

### Review method

This systematic review (SR) protocol has been registered on PROSPERO (CRD42022352095). The protocol will follow the Preferred Reporting Items for Systematic Reviews and Meta-Analyses Protocol (PRISMA-P) guidelines (16), according to the PRISMA-P checklist (Supplementary Appendix 1). SR will be conducted based on PRISMA-NMA and A Measure Tool to Assess Systematic Reviews-2 guidelines (17, 18). It is scheduled to begin on 1 October 2022 and end on 1 December 2022.

### Patient and public involvement

The study will not involve direct participant participation. Only randomized controlled trial (RCT) data from the databases will be used.

### Eligibility criteria

#### Types of studies

Randomized controlled trials on non-pharmacological therapies of BPSD, regardless of language or publication type, will be considered.

#### Types of participants

There will be no limitations on participants’ gender, age, or ethnicity. Regardless of the subtype or method of diagnosis, individuals must be diagnosed with dementia. Any physical or psychiatric comorbidities are allowed.

#### Types of interventions

Non-pharmacological interventions such as physical exercise (19, 20), music therapy (21), aromatherapy therapy (22), reminiscence therapy (23), cognitive interventions (24), light therapy (25), massage therapies (26), non-invasive brain stimulation (27), acupuncture therapy (28), etc. will be included.

#### Types of control group

Different non-pharmacological therapies will form the basis for the control group, which will include the placebo group, waiting-list group, treatment as usual group, conventional-based medicine group, etc.

#### Exclusion criteria

Exclusion criteria for studies are as follows:

1. Non-randomized clinical studies, quasi-RCTs, cluster RCTs, case studies, qualitative studies, conference abstracts, animal studies, letters, comments, and duplicated articles.
2. A lack of research information.

### Information sources and search strategy

Studies published since inception to 1 October 2022 will be retrieved from the following databases: CNKI, WF, VIP Database, SinoMed, Web of Science (WOS), Embase, PubMed, PsycINFO, the CENTRAL, and AMED. To minimize publication bias, we will also retrieve data from clinical registries (WHO ICTRP, Clinical trials.gov, and ChiCTR). Furthermore, we will manually search the included papers’ references and similar published SRs for related studies.

Each database search strategy is shown in Supplementary Appendix 2. The following search terms will be used: (1) disease: dementia, behavioral and psychological symptoms of dementia, etc.; (2) non-pharmacological intervention: physical exercise, music therapy, reminiscence therapy, cognitive stimulation, etc.; and (3) study types: randomized controlled trials, or RCTs. Similar retrieval strategies will be used for other electronic databases. The terms will be used alone or in combination with “and” and “or.”

## Study records

### Study selection and data extraction

Two methodologically trained reviewers (Y-QL and Z-HY) will screen titles, keywords, and abstracts for relevance. Duplicate and ineligible studies will be excluded from the review process. All remaining studies will be examined for inclusion once the full text has been reviewed. In case of disagreements, a third reviewer (F-RL or L-XJ) will countercheck and arbitrate.

**FIGURE 1:**
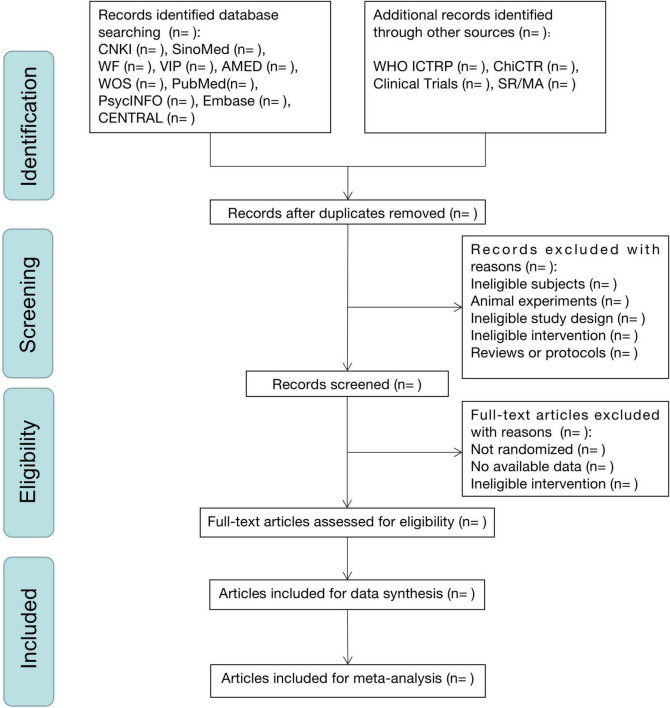
Flow chart of the selection process.

Two independent investigators (Y-QL and Z-HY) will extract data using a pre-designed extraction form including (1) identification information (i.e., year, country, and authors); (2) general information (i.e., sample size, study design, and allocation ratio); (3) participants (i.e., age, sex, race/ethnicity, trial inclusion and exclusion criteria, dementia type and severity, and comorbidity); (4) interventions and control; (5) outcomes; and (6) main results. We will contact respective authors concerning missing data, if any. Figure 1 depicts the entire study selection process.

### Types of outcome measures

#### Primary outcomes

The primary outcome is the severity of BPSD assessed using the Neuropsychiatric Inventory (NPI) (29). The NPI is a commonly used, objective and sensitive tool for assessing the effectiveness of treatment. It includes 12 neuropsychiatric symptoms typical of dementia: delusions, hallucinations, depression, anxiety, apathy, euphoria, agitation or aggression, aberrant motor activity, sleep disturbances, eating disorders, and disinhibition.

#### Secondary outcomes

(i) Depression scores will be calculated using the Cornell Scale for Depression in Dementia (CSDD). It is widely used to assess depressive symptoms in patients with dementia. It is administered through interviews with patients and caregivers (30). The CSDD consists of 19 items divided into five subscales (mood, behavioral disturbances, physical signs, cyclic functions, and ideational disturbances). The score ranges from 0 to 38, with higher scores indicating higher levels of depression.
(ii) The Cohen-Mansfield Agitation Inventory (CMAI) will be used to evaluate agitation. It is one of the most commonly used scales to evaluate behavioral symptoms of dementia and is focused on measuring agitation and aggression (31). It consists of 29 items divided into 3 subscales: physical aggression, physical non-aggression (e.g., pacing and wandering), and verbally aggressive behavior. Each item will be rated on a scale of 1 to 5, with a total score ranging from 14 to 70. Agitation is more pronounced with higher scores.
(iii) Apathy will be measured using the Apathy Evaluation Scale-Clinical version (AES-C). Clinicians often use this scale to assess and quantify the emotional, behavioral, and cognitive domains of apathy (32). The scale consists of 18 questions with a Likert scale ranging from 1 to 4 and total scores ranging from 18 to 72. Higher scores indicate higher levels of apathy.
(iv) Activities of daily living will be measured using the Abilities of Daily Living (ADL) scale (33). The ADL is one of the most used assessment tools to quantify physical functional capacity. In the ADL scale, there are 10 questions related to non-instrumental activities of daily living and 5 questions related to instrumental activities of daily living.
(v) Quality of life will be assessed using the Quality of Life in Alzheimer’s Disease (Qol-AD) scale (34). The Qol-AD is commonly used to assess the quality of life in people with dementia and is indicated for mild to severe dementia patient evaluation. There are 13 items scored using a 4-point Likert scale, with higher scores indicating better quality of life. Previous studies have shown that the scale can demonstrate sensitivity to psychosocial interventions (35).
(vi) Adverse events associated with intervention approaches will be described directly in order to assess safety.

### Quality assessment

Two assessors (Y-QL and Z-HY) will independently appraise the quality of the selected RCTs using the Risk of Bias Assessment Tool (Rob 2.0) from the Cochrane Collaboration (36). There are five types of bias covered by this tool: (1) the randomization process; (2) deviations from intended interventions; (3) missing outcomes data; (4) outcome measurement; and (5) selection of the reported results. The included trials will be rated and classified as low risk, high risk, or some concerns. If all domains were at low risk, we judged the study results to have an overall low risk of bias, some concerns if there were some concerns in any domain, and a high risk of bias if we judged any domain to be at high risk. A third reviewer (F-RL or L-XJ) will be consulted during the final decision-making process. Graphs will be generated using the Shiny app.^1^

### Analysis

#### Pairwise meta-analysis

The pairwise meta-analysis will be performed using STATA 15.0 (StataCorp LP). As outcomes, we will use pre-post differences or endpoint scores for the included RCTs. The relative risk (RR) of dichotomous data will be presented with a 95% confidence interval (CI), while the weighted mean differences (WMD) of continuous data will be presented with 95% CI. The statistical heterogeneity will be identified and measured by *I*^2^ statistics and *p*-value. A fixed-effects model will be applied if *p* > 0.1 and *I*^2^ < 50%; otherwise, the random effects model will be used (37).

#### Network meta-analysis

We will use the Aggregate Data Drug Information System (V.1.16.8, Drugis, Groningen, Netherlands) and Markov Chain Monte Carlo (MCMC) algorithm to perform Bayesian network analysis (38). Further, STATA V15.0 will be used for generating the network plots for each treatment comparison. The rankings will then be generated for a variety of non-pharmacological interventions. Comparisons between interventions will be presented as a network plot, and rank plots will be used to illustrate the contribution of different designs to the final effect size of NMA (39). Moreover, the local inconsistency at the network level will also be assessed using the node splitting model, and based on the results, the consistency or inconsistency model will be selected. A statistically significant difference between indirect and direct multiple treatment comparisons is present if *p* < 0.05. To evaluate the convergence of the results, we will analyze the potential scale reduction factor (PSRF), with a PSRF value close to 1 indicating successful convergence (40).

#### Subgroup analysis and sensitivity analysis

Based on the guidelines from Cochrane Handbook 5.4, we consider heterogeneity as significant when *I*^2^ ≥ 50% and *p* < 0.05 (41). If there is significant heterogeneity, we plan to perform a subgroup analysis based on the type of intervention (e.g., exercise, training, therapeutic, music, etc.). Meanwhile, a sensitivity analysis will be conducted to ensure accuracy and stability of inferences from our results to remove the effects of small sample-sized trials and high methodological risk of bias.

### Assessment of publication bias

The comparison-adjusted funnel plot will be used if more than ten trials are included to visualize potential publication bias. Ideally, the data from these studies would be represented by a symmetrical inverted funnel plot. An asymmetrical funnel plot indicates the presence of publication bias.

### Quality of evidence

Grading of Recommendations Assessment, Development, and Evaluation (GRADE) system will be used to assess the quality of evidence (42). Evidence quality will be ranked as “high,” “moderate,” “low,” or “critically low.” It may be downgraded due to the risk of bias, inconsistency, indirectness, imprecision, and publication bias. Evidence may be upgraded due to a large magnitude of effect, a dose-response gradient, or attenuation by plausible confounding.

## Discussion

There is often great concern about cognitive decline when an individual develops dementia. Numerous non-pharmacological interventions, such as physical activity interventions, cognitive training, cognitive stimulation, and combinations of these interventions, have been proven effective in improving cognitive function in patients with dementia (43–46). However, in addition to cognitive decline, the high prevalence of BPSD is another significant issue for patients, as well as their families and caregivers, seriously affecting their quality of life. A number of systematic reviews and meta-analyses have demonstrated that aerobic exercise and acupuncture treatment are effective in treating BPSD (28, 47). Moreover, two reviews uncovered that massage therapy, activities and music therapy could significantly improve agitation in people with dementia (48, 49). Nonetheless, most previous meta-analyses focused on the efficacy of interventions on a single BPSD symptom or the overall change in BPSD with a single non-pharmacological therapy. There is still a lack of comprehensive and stronger evidence-based medical evidence that can be used to compare which type of intervention is most effective.

Network meta-analysis allows for the comparison of multiple interventions to obtain an efficacy ranking based on direct and indirect comparisons (50), allowing for a more precise effect size estimation. The present study will examine the effects of various non-pharmacological interventions on overall BPSD, agitation, depression, and apathy symptoms while ranking the efficacy of different treatments for BPSD. Collectively, our findings may lead to the identification of optimal therapeutic strategies for BPSD and may guide future research and clinical practice.

### Amendments

PROSPERO registration will include protocol amendments. We will document and publish any modifications to this protocol along with the results of the systematic review.

## Author’s note

Studies published since inception to 1 October 2022 will be retrieved from each databases.

In December 2022, this study will be completed, and the timeline will be as follows:

– Complete the data extract by the end of November 2022.
– Complete the data analysis and the manuscript writing by the end of December 2022.

## Ethics statement

Every aspect of the work must be examined and resolved by the authors to ensure its accuracy and integrity. As no private or confidential patient data will be included, ethical clearance is not required. The privacy and rights of the individual will not be compromised.

## Author contributions

Y-QL, Z-HY, L-XJ, and F-RL: conception and design. Y-QL and Z-HY: collection and assembly of data, data analysis, and interpretation. All authors wrote of the manuscript and approved the submitted version.

## Footnotes

1
https://mcguinlu.shinyapps.io/robvis/
