# Supplementary material for: Non-pharmacological interventions for behavioral and psychological symptoms of dementia: A systematic review and network meta-analysis protocol
Source: Front Psychiatry. 2022 Nov 29;13:1039752. doi: 10.3389/fpsyt.2022.1039752 (PMC9744934; doi:10.3389/fpsyt.2022.1039752)
Supplement: Supplementary file 2 [file Data_Sheet_2.docx]

**Appendix 2. Search strategies of each database.**

**PubMed**

#1 "behavioral and psychological symptoms of dementia" [Title/Abstract] OR "BPSD" [Title/Abstract] OR "behav* psychological symptoms dementia" [Title/Abstract] OR "challenging behav*" [Title/Abstract] OR "disturbing behav*" [Title/Abstract] OR "difficult behav*" [Title/Abstract] OR "disruptive behav*" [Title/Abstract] OR "behav* concern" [Title/Abstract] OR "agitat*" [Title/Abstract] OR "restles*" [Title/Abstract] OR "pacing" [Title/Abstract] OR "resist*" [Title/Abstract] OR "apathy" [Title/Abstract] OR "social* disinhibiti*" [Title/Abstract] OR "sexual* disinhibiti*" [Title/Abstract] OR "catastrophic reaction" [Title/Abstract] OR "verbal outbursts" [Title/Abstract] OR "screaming" [Title/Abstract] OR "delusion" [Title/Abstract] OR "hallucination" [Title/Abstract] OR "anxiety" [Title/Abstract] OR "depression" [Title/Abstract] OR "neuropsychiatr* symptoms" [Title/Abstract]

#2 "Dementia"[Mesh]

#3 "alzheimer* disease"[Title/Abstract] OR "lewy body dementia"[Title/Abstract] OR "vascular dementia"[Title/Abstract] OR "organic brain disease"[Title/Abstract] OR "organic brain syndrome"[Title/Abstract] OR "dementia in Parkinson’s disease"[Title/Abstract] OR "frontotemporal dementia"[Title/Abstract]

#4 #2 OR #3

#5 "nonpharmacological management"[Mesh] OR "nonpharmacological intervention"[Mesh] OR "nonpharmacological treatment"[Mesh]

#6 "physical exercise"[Title/Abstract] OR "music therapy"[Title/Abstract] OR "aromatherapy therapy"[Title/Abstract] OR "reminiscence therapy"[Title/Abstract] OR "cognitive stimulation therapy"[Title/Abstract] OR "cognitive rehabilitation"[Title/Abstract] OR "computerized-based training"[Title/Abstract] OR "psychosocial therapy"[Title/Abstract] OR "light therapy"[Title/Abstract] OR "massage therapies"[Title/Abstract] OR "non-invasive brain stimulation"[Title/Abstract] OR "acupuncture therapy"[Title/Abstract]

#7 #5 OR #6

#8 "clinical trials, randomized"[Mesh Terms] OR "controlled clinical trials, randomized"[Mesh Terms] OR "clinical trials as topic"[MeSH Terms] OR "random allocation"[MeSH Terms] OR "therapeutic use"[MeSH Subheading]

#9 "clinical"[Title/Abstract] AND "trial"[Title/Abstract]) OR "clinical trial"[Publication Type] OR "random*"[Title/Abstract]

#10 #8 OR #9

#11 #1 AND #4 AND #7 AND #10

**EMBASE via Elsevier**

#1 'behavioral and psychological symptoms of dementia'/exp

#2 'BPSD':ti,ab,kw OR 'behav* psychological symptoms dementia':ti,ab,kw OR 'challenging behav*':ti,ab,kw OR 'disturbing behav*':ti,ab,kw OR 'difficult behav*':ti,ab,kw OR 'disruptive behav*':ti,ab,kw OR 'behav* concern':ti,ab,kw OR 'agitat*':ti,ab,kw OR 'restles*':ti,ab,kw OR 'pacing':ti,ab,kw OR 'resist*':ti,ab,kw OR 'apathy':ti,ab,kw OR 'social* disinhibiti*':ti,ab,kw OR 'sexual* disinhibiti*':ti,ab,kw OR 'catastrophic reaction':ti,ab,kw OR 'verbal outbursts':ti,ab,kw OR 'screaming':ti,ab,kw OR 'delusion':ti,ab,kw OR 'hallucination':ti,ab,kw OR 'anxiety':ti,ab,kw OR 'depression':ti,ab,kw OR 'neuropsychiatr* symptoms':ti,ab,kw

#3 #1 OR #2

#4 'dementia'/exp OR 'Alzheimer disease'/exp OR 'Lewy body'/exp OR 'Parkinson disease'/exp

#5 'dement*':ti,ab,kw OR 'Alzheimer*':ti,ab,kw OR 'Lewy body':ti,ab,kw OR 'Parkinson':ti,ab,kw OR 'vascular dementia':ti,ab,kw OR 'organic brain disease':ti,ab,kw OR 'frontotemporal dementia':ti,ab,kw

#6 #4 OR #5

#7 'nonpharmacological management'/exp OR 'nonpharmacological intervention'/exp OR 'nonpharmacological treatment'/exp

#8 'physical exercise':ti,ab,kw OR 'music therapy':ti,ab,kw OR 'aromatherapy therapy':ti,ab,kw OR 'reminiscence therapy':ti,ab,kw OR 'cognitive stimulation therapy':ti,ab,kw OR 'cognitive rehabilitation':ti,ab,kw OR 'computerized-based training':ti,ab,kw OR 'psychosocial therapy':ti,ab,kw OR 'light therapy':ti,ab,kw OR 'massage therapies':ti,ab,kw OR 'non-invasive brain stimulation':ti,ab,kw OR 'acupuncture therapy':ti,ab,kw

#9 #7 OR #8

#10 'randomized controlled trial':ti,ab,kw OR 'randomized clinical trial':ti,ab,kw

#11 #3 AND #6 AND #9 AND #10

**PsychINFO via EBSCO**

#1 SU ( behavioral and psychological symptoms of dementia ) OR TX BPSD OR TX behav* psychological symptoms dementia OR TX challenging behav* OR TX disturbing behav* OR TX difficult behav* OR TX disruptive behav* OR TX behav* concern OR TX agitat* OR TX restles* OR TX pacing OR TX resist* OR TX apathy OR TX social* disinhibiti* OR TX sexual* disinhibiti* OR TX catastrophic reaction OR TX verbal outbursts OR TX screaming OR TX delusion OR TX hallucination OR TX anxiety OR TX depression OR TX neuropsychiatr* symptoms

#2 SU Dementia OR TX dement* OR TX Alzheimer* OR TX Lewy body OR TX vascular dementia* OR TX Parkinson* OR TX organic brain disease OR TX frontotemporal dementia

#3 SU nonpharmacological management OR SU nonpharmacological intervention OR SU nonpharmacological treatment OR TX physical exercise OR TX music therapy OR TX aromatherapy therapy OR TX reminiscence therapy OR TX cognitive stimulation therapy OR TX cognitive rehabilitation OR TX computerized-based training OR TX light therapy OR TX massage therapies OR TX non-invasive brain stimulation OR TX acupuncture therapy

#4 TX randomized controlled trial OR TX randomized clinical trial

#5 #1 AND #2 AND #3 AND #4

**Web of science**

TS=((behavioral and psychological symptoms of dementia OR BPSD OR behav* psychological symptoms dementia OR challenging behav* OR disturbing behav* OR difficult behav* OR disruptive behav* OR behav* concern OR agitat* OR restles* OR pacing OR resist* OR apathy OR social* disinhibiti* OR sexual* disinhibiti* OR catastrophic reaction OR verbal outbursts OR screaming OR delusion OR hallucination OR anxiety OR depression OR neuropsychiatr* symptoms) AND (dement* OR Alzheimer* OR Lewy body OR Parkinson OR vascular dementia OR organic brain disease OR frontotemporal dementia) AND (nonpharmacological management OR nonpharmacological intervention OR nonpharmacological treatment OR physical exercise OR music therapy OR aromatherapy therapy OR reminiscence therapy OR cognitive stimulation therapy OR cognitive rehabilitation OR computerized-based training OR psychosocial therapy OR light therapy OR massage therapies OR non-invasive brain stimulation OR acupuncture therapy) AND (randomized controlled trial OR randomized clinical trial))

**CENTRAL**

#1 behavioral and psychological symptoms of dementia:ti,ab,kw OR BPSD:ti,ab,kw OR behav* psychological symptoms dementia:ti,ab,kw OR challenging behav*:ti,ab,kw OR disturbing behav*:ti,ab,kw OR difficult behav*:ti,ab,kw OR disruptive behav*:ti,ab,kw OR behav* concern:ti,ab,kw OR agitat*:ti,ab,kw OR restles*:ti,ab,kw OR pacing:ti,ab,kw OR resist*:ti,ab,kw OR apathy:ti,ab,kw OR social* disinhibiti*:ti,ab,kw OR sexual* disinhibiti*:ti,ab,kw OR catastrophic reaction:ti,ab,kw OR verbal outbursts:ti,ab,kw OR screaming:ti,ab,kw OR delusion:ti,ab,kw OR hallucination:ti,ab,kw OR anxiety:ti,ab,kw OR depression:ti,ab,kw OR neuropsychiatr* symptoms:ti,ab,kw

#2 Mesh descriptor: [Dementia] explode all trees;

#3 dement*:ti,ab,kw OR Alzheimer*:ti,ab,kw OR Lewy body:ti,ab,kw OR Parkinson:ti,ab,kw OR vascular dementia:ti,ab,kw OR organic brain disease:ti,ab,kw OR frontotemporal dementia:ti,ab,kw

#4 #2 OR #3

#5 Mesh descriptor: [nonpharmacological management] explode all trees

#6 Mesh descriptor: [nonpharmacological intervention] explode all trees

#7 Mesh descriptor: [nonpharmacological treatment] explode all trees

#8 physical exercise:ti,ab,kw OR music therapy:ti,ab,kw OR aromatherapy therapy:ti,ab,kw OR reminiscence therapy:ti,ab,kw OR cognitive stimulation therapy:ti,ab,kw OR cognitive rehabilitation:ti,ab,kw OR computerized-based training:ti,ab,kw OR psychosocial therapy:ti,ab,kw OR light therapy:ti,ab,kw OR massage therapies:ti,ab,kw OR non-invasive brain stimulation:ti,ab,kw OR acupuncture therapy:ti,ab,kw

#9 #5 OR #6 OR #7 OR #8

#10 randomized controlled trial:ti,ab,kw OR randomized clinical trial:ti,ab,kw

#11 #1 AND #4 AND #9 AND #10

**AMED via EBSCO**

#1 SU ( behavioral and psychological symptoms of dementia ) OR TX BPSD OR TX behav* psychological symptoms dementia OR TX challenging behav* OR TX disturbing behav* OR TX difficult behav* OR TX disruptive behav* OR TX behav* concern OR TX agitat* OR TX restles* OR TX pacing OR TX resist* OR TX apathy OR TX social* disinhibiti* OR TX sexual* disinhibiti* OR TX catastrophic reaction OR TX verbal outbursts OR TX screaming OR TX delusion OR TX hallucination OR TX anxiety OR TX depression OR TX neuropsychiatr* symptoms

#2 SU Dementia OR TX dement* OR TX Alzheimer* OR TX Lewy body OR TX vascular dementia* OR TX Parkinson* OR TX organic brain disease OR TX frontotemporal dementia

#3 SU nonpharmacological management OR SU nonpharmacological intervention OR SU nonpharmacological treatment OR TX physical exercise OR TX music therapy OR TX aromatherapy therapy OR TX reminiscence therapy OR TX cognitive stimulation therapy OR TX cognitive rehabilitation OR TX computerized-based training OR TX light therapy OR TX massage therapies OR TX non-invasive brain stimulation OR TX acupuncture therapy

#4 TX randomized controlled trial OR TX randomized clinical trial

#5 #1 AND #2 AND #3 AND #4

**CNKI**

SU=('痴呆的精神行为症状'+'BPSD'+'行为异常'+'精神异常'+'神经精神症状'+'幻觉'+'妄想'+'攻击行为'+'激越'+'抑郁'+'焦虑'+'失眠'+'睡眠障碍'+'不寐'+'忧虑'+'冷漠'+'淡漠'+'易怒'+'流浪') AND SU=('痴呆'+'老年痴呆'+'阿尔茨海默'+'阿尔兹海默'+'路易体痴呆'+'血管性痴呆'+'帕金森'+'脑器质性疾病'+'额颞叶痴呆') AND SU=('非药物疗法'+'非药物干预'+'非药物手段'+'非药物管理'+'运动'+'光'+'音乐'+'芳香'+'脑刺激'+'认知训练'+'回忆'+'认知康复'+'计算机训练'+'针灸'+'按摩'+'推拿'+'艾灸'+'针') AND (TKA=(‘随机’+‘对照’) OR SU=(‘随机对照试验’))

**WF**

(题名或关键词:(痴呆的精神行为症状 OR BPSD OR 行为异常 OR 精神异常 OR 神经精神症状 OR 幻觉OR 妄想 OR 攻击行为 OR 激越 OR 抑郁 OR 焦虑 OR 失眠 OR 睡眠障碍OR 不寐 OR 忧虑 OR 冷漠 OR 淡漠 OR 易怒 OR 流浪)) and (题名或关键词:(痴呆 OR 老年痴呆 OR 阿尔茨海默 OR 阿尔兹海默症 OR 路易体痴呆 OR 血管性痴呆 OR 帕金森 OR 脑器质性疾病 OR 额颞叶痴呆)) and (题名或关键词:(非药物疗法 OR 非药物干预 OR 非药物手段 OR 非药物管理 OR 运动 OR 光 OR 音乐 OR 芳香 OR 脑刺激 OR 认知训练 OR 回忆 OR 认知康复OR 计算机训练 OR 针灸 OR 按摩 OR 推拿 OR 艾灸 OR 针)) and (题名或关键词:(随机 OR 对照) OR 主题:(随机对照试验))

**Chongqing VIP**

M=(痴呆的精神行为症状 OR BPSD OR 行为异常 OR 精神异常 OR 神经精神症状 OR 幻觉OR 妄想 OR 攻击行为 OR 激越 OR 抑郁 OR 焦虑 OR 失眠 OR 睡眠障碍OR 不寐 OR 忧虑 OR 冷漠 OR 淡漠 OR 易怒 OR 流浪) and M=(痴呆 OR 老年痴呆 OR 阿尔茨海默 OR 阿尔兹海默 OR 路易体痴呆 OR 血管性痴呆 OR 帕金森 OR 脑器质性疾病 OR 额颞叶痴呆) and M=(非药物疗法 OR 非药物干预 OR 非药物手段 OR 非药物管理 OR 运动 OR 光 OR 音乐 OR 芳香 OR 脑刺激 OR 认知训练 OR 回忆 OR 认知康复OR 计算机训练 OR 针灸 OR 按摩 OR 推拿 OR 艾灸 OR 针) and R=(随机 OR 对照)

**SinoMed**

1 "痴呆的精神行为症状"[常用字段:智能] OR "BPSD"[常用字段:智能] OR "行为异常"[常用字段:智能] OR "精神异常"[常用字段:智能] OR "神经精神症状"[常用字段:智能] OR "幻觉"[常用字段:智能] OR "妄想"[常用字段:智能] OR "攻击行为"[常用字段:智能] OR "激越"[常用字段:智能] OR "抑郁"[常用字段:智能] OR "焦虑"[常用字段:智能] OR "失眠"[常用字段:智能] OR "睡眠障碍"[常用字段:智能] OR "不寐"[常用字段:智能] OR "忧虑"[常用字段:智能] OR "冷漠"[常用字段:智能] OR "淡漠"[常用字段:智能] OR "易怒"[常用字段:智能] OR "流浪"[常用字段:智能]

2 "痴呆"[加权:扩展]

3 "老年痴呆"[常用字段:智能] OR "阿尔茨海默"[常用字段:智能] OR "阿尔兹海默"[常用字段:智能] OR "路易体痴呆"[常用字段:智能] OR "血管性痴呆"[常用字段:智能] OR "帕金森"[常用字段:智能] OR "脑器质性疾病"[常用字段:智能] OR "额颞叶痴呆"[常用字段:智能]

4 2 OR 3

5 "非药物疗法"[常用字段:智能] OR "非药物干预"[常用字段:智能] OR "非药物手段"[常用字段:智能] OR "非药物管理"[常用字段:智能] OR "运动"[常用字段:智能] OR "音乐"[常用字段:智能] OR "芳香"[常用字段:智能] OR "脑刺激"[常用字段:智能] OR "光"[常用字段:智能] OR "认知训练"[常用字段:智能] OR "认知康复"[常用字段:智能] OR "计算机训练"[常用字段:智能] OR "回忆"[常用字段:智能] OR "针灸"[常用字段:智能] OR "按摩"[常用字段:智能] OR "推拿"[常用字段:智能] OR "艾灸"[常用字段:智能] OR "针"[常用字段:智能]

6 "随机对照试验"[不加权:扩展]

7 "随机"[常用字段:智能] OR "对照"[常用字段:智能]

8 6 OR 7

9 1 AND 4 AND 5 AND 8
